# Counties with High COVID-19 Incidence and Relatively Large Racial and Ethnic Minority Populations — United States, April 1–December 22, 2020

**DOI:** 10.15585/mmwr.mm7013e1

**Published:** 2021-04-02

**Authors:** Florence C. Lee, Laura Adams, Sierra J. Graves, Greta M. Massetti, Renee M. Calanan, Ana Penman-Aguilar, S. Jane Henley, Francis B. Annor, Michelle Van Handel, Noah Aleshire, Tonji Durant, Jennifer Fuld, Sean Griffing, Laura Mattocks, Leandris Liburd

**Affiliations:** ^1^CDC COVID-19 Response Team; ^2^Oak Ridge Institute for Science and Education, Oak Ridge, Tennessee.

Long-standing systemic social, economic, and environmental inequities in the United States have put many communities of color (racial and ethnic minority groups) at increased risk for exposure to and infection with SARS-CoV-2, the virus that causes COVID-19, as well as more severe COVID-19–related outcomes (*1*–*3*). Because race and ethnicity are missing for a proportion of reported COVID-19 cases, counties with substantial missing information often are excluded from analyses of disparities (*4*). Thus, as a complement to these case-based analyses, population-based studies can help direct public health interventions. Using data from the 50 states and the District of Columbia (DC), CDC identified counties where five racial and ethnic minority groups (Hispanic or Latino [Hispanic], non-Hispanic Black or African American [Black], non-Hispanic Asian [Asian], non-Hispanic American Indian or Alaska Native [AI/AN], and non-Hispanic Native Hawaiian or other Pacific Islander [NH/PI]) might have experienced high COVID-19 impact during April 1–December 22, 2020. These counties had high 2-week COVID-19 incidences (>100 new cases per 100,000 persons in the total population) and percentages of persons in five racial and ethnic groups that were larger than the national percentages (denoted as “large”). During April 1–14, a total of 359 (11.4%) of 3,142 U.S. counties reported high COVID-19 incidence, including 28.7% of counties with large percentages of Asian persons and 27.9% of counties with large percentages of Black persons. During August 5–18, high COVID-19 incidence was reported by 2,034 (64.7%) counties, including 92.4% of counties with large percentages of Black persons and 74.5% of counties with large percentages of Hispanic persons. During December 9–22, high COVID-19 incidence was reported by 3,114 (99.1%) counties, including >95% of those with large percentages of persons in each of the five racial and ethnic minority groups. The findings of this population-based analysis complement those of case-based analyses. In jurisdictions with substantial missing race and ethnicity information, this method could be applied to smaller geographic areas, to identify communities of color that might be experiencing high potential COVID-19 impact. As areas with high rates of new infection change over time, public health efforts can be tailored to the needs of communities of color as the pandemic evolves and integrated with longer-term plans to improve health equity. 

To assess potential COVID-19 impact on racial and ethnic minority groups, CDC examined two population-based measures: incidence of COVID-19 at the county level during three successive 2-week periods during April 1–December 22, 2020, and the percentage of the county population accounted for by each racial and ethnic minority group. Two-week COVID-19 incidence was calculated as numbers of cases (*5*) per 100,000 persons collected from state and local health department websites*; counties with >100 new cases per 100,000 persons during the 2-week period were considered to have high incidence.

The percentage of county population represented by five racial and ethnic minority groups was calculated using 2019 U.S. Census population estimates.^†^ Counties whose percentage of racial and ethnic minority persons exceeded the 2019 national percentage were considered to have relatively large populations of the respective racial and ethnic minority group. For the Hispanic, Black, Asian, AI/AN, and NH/PI groups, these were percentages in excess of 18.5%, 12.5%, 5.8%, 0.7%, and 0.2%, respectively. Whereas the term “population” is used to describe all persons within a reported race and ethnicity category, the diversity of backgrounds and experiences that exists within these broad groups is recognized.

Counties were considered to have high potential COVID-19 impact during the 2-week period for a racial and ethnic minority group if they had both high COVID-19 incidence and a large population of the respective group. Counties were considered to have relatively low potential COVID-19 impact during the 2-week period for a racial and ethnic minority group if they had a large population of the respective group and low COVID-19 incidence or had a small population of the respective group, regardless of COVID-19 incidence. To illustrate where counties with high potential COVID-19 impact were located for each racial and ethnic minority group, maps were created for three time periods, representing the beginning (April 1–14), middle (August 5–18), and end (December 9–22) of the analysis period (Supplementary Figures 1–3, https://stacks.cdc.gov/view/cdc/104229). In these maps, high COVID-19 incidence was further categorized as >100 to ≤500 and >500 new cases per 100,000 persons. Large population of the respective racial and ethnic minority group was further categorized as >national percentage to ≤upper cutpoint and >upper cutpoint, where the upper cutpoint was determined using Jenks natural breaks.^§^ The four U.S. Census regions were used to describe groups of counties for interpretation.^¶^

During April 1–14, high COVID-19 incidence was reported by 359 (11.4%) counties, most of which were in the Northeast and South (Figure 1). High COVID-19 incidence was reported by 28.7%, 27.9%, 12.5%, 5.1%, and 0.6% of counties with large Asian, Black, Hispanic, AI/AN, and NH/PI populations, respectively, during this period (Table) (Figure 2) (Supplementary Figure 1, https://stacks.cdc.gov/view/cdc/104229).

**FIGURE 1:**
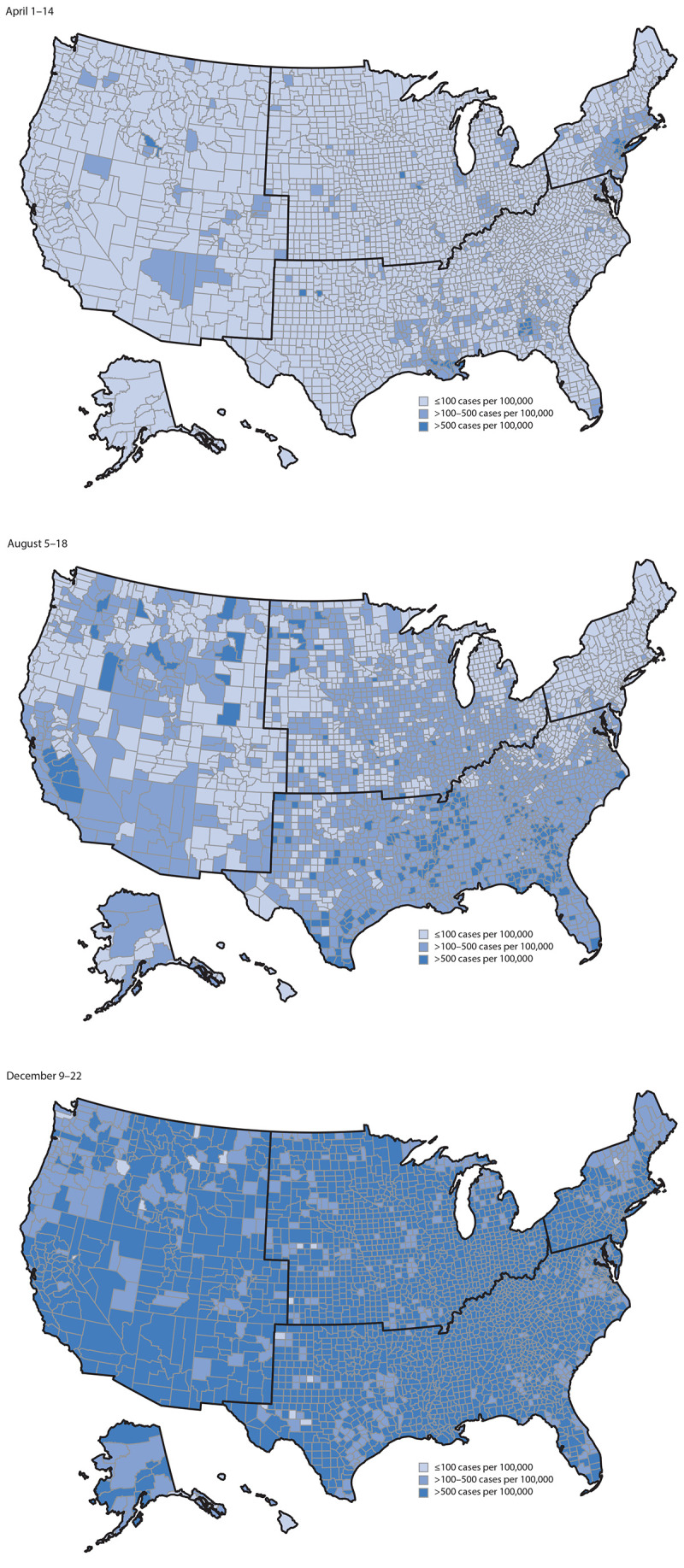
Counties with high COVID-19 incidence,* by county for April 1–14, August 5–18, and December 9–22 — United States,^†^ April 1–December 22, 2020 * >100 new cases per 100,000 persons in the 2-week period. † U.S. Census regions are outlined in black.

**TABLE:** Percentage of counties with high COVID-19 incidence* and large population percentages of five racial and ethnic minority groups^†^ — United States, April 1–14, August 5–18, and December 9–22, 2020

COVID-19 incidence* (% county population)^†^	No. (%) of counties		
	Apr 1–14	Aug 5–18	Dec 9–22
**Counties with large Hispanic or Latino populations (>18.5%)**	439 (100)	439 (100)	439 (100)
>500 cases per 100,000 (>46.0%–96.4%)	1 (0.2)	27 (6.2)	101 (23.0)
>100–500 cases per 100,000 (>46.0–96.4%)	5 (1.1)	68 (15.5)	16 (3.6)
>500 cases per 100,000 (>18.5–46.0%)	15 (3.4)	23 (5.2)	270 (61.5)
>100–500 cases per 100,000 (>18.5–46.0%)	34 (7.7)	209 (47.6)	48 (10.9)
**Counties with large Black, non-Hispanic populations (>12.5%)**	681 (100)	681 (100)	681 (100)
>500 cases per 100,000 (>37.0–85.9%)	12 (1.8)	53 (7.8)	184 (27.0)
>100–500 cases per 100,000 (>37.0–85.9%)	73 (10.7)	156 (22.9)	30 (4.4)
>500 cases per 100,000 (>12.5–37.0%)	10 (1.5)	75 (11.0)	394 (57.9)
>100–500 cases per 100,000 (>12.5–37.0%)	95 (14.0)	345 (50.7)	73 (10.7)
**Counties with large Asian, non-Hispanic populations (>5.8%)**	136 (100)	136 (100)	136 (100)
>500 cases per 100,000 (>17.7–42.8%)	2 (1.5)	1 (0.7)	10 (7.4)
>100–500 cases per 100,000 (>17.7–42.8%)	1 (0.7)	10 (7.4)	8 (5.9)
>500 cases per 100,000 (>5.8–17.7%)	10 (7.4)	3 (2.2)	85 (62.5)
>100–500 cases per 100,000 (>5.8–17.7%)	26 (19.1)	74 (54.4)	28 (20.6)
**Counties with large AI/AN, non-Hispanic populations (>0.7%)**	826 (100)	826 (100)	826 (100)
>500 cases per 100,000 (>30.3%–90.4%)	0	5 (0.6)	33 (4.0)
>100–500 cases per 100,000 (>30.3%–90.4%)	5 (0.6)	21 (2.5)	7 (0.8)
>500 cases per 100,000 (>0.7%–30.3%)	3 (0.4)	37 (4.5)	602 (72.9)
>100–500 cases per 100,000 (>0.7%–30.3%)	34 (4.1)	364 (44.1)	171 (20.7)
**Counties with large NH/PI, non-Hispanic populations (>0.2%)**	173 (100)	173 (100)	173 (100)
>500 cases per 100,000 (>11.8%–48.8%)	0	0	0
>100–500 cases per 100,000 (>11.8%–48.8%)	0	0	0
>500 cases per 100,000 (>0.2%–11.8%)	0	8 (4.6)	118 (68.2)
>100–500 cases per 100,000 (>0.2%–11.8%)	1 (0.6)	106 (61.3)	47 (27.2)

**Abbreviations**: AI/AN = American Indian or Alaska Native; NH/PI = Native Hawaiian or other Pacific Islander.

* >100 new cases per 100,000 persons in the 2-week period. High COVID-19 incidence was further categorized as >100 to ≤500 new cases per 100,000 persons and >500 new cases per 100,000 persons.

^†^ Percentage of racial and ethnic minority group populations in the county higher than the national percentages: 12.5% (non-Hispanic Black), 18.5% (Hispanic/Latino), 5.8% (non-Hispanic Asian), 0.7% (non-Hispanic AI/AN), and 0.2% (non-Hispanic NH/PI). Large population was further categorized as >national percentage to ≤upper cutpoint and >upper cutpoint, where the upper cutpoint was determined using Jenks natural breaks.

**FIGURE 2:**
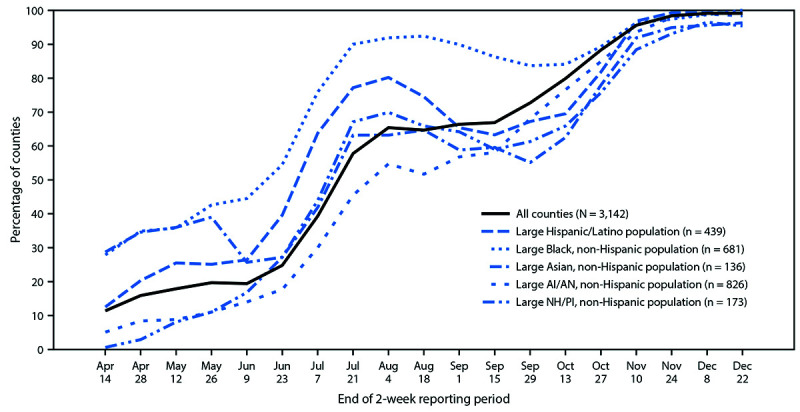
Percentage of counties with high COVID-19 incidence* among U.S. counties with large population percentages of five racial and ethnic minority groups^†^ — United States, April 1–December 22, 2020 **Abbreviations**: AI/AN = American Indian/Alaska Native; NH/PI = Native Hawaiian or other Pacific Islander. * >100 new cases per 100,000 persons in the 2-week period. † Percentage of racial and ethnic minority group populations in the county higher than the national percentages: 12.5% (non-Hispanic Black), 18.5% (Hispanic/Latino), 5.8% (non-Hispanic Asian), 0.7% (non-Hispanic AI/AN), and 0.2% (non-Hispanic NH/PI).

As the geographic distribution of counties reporting high COVID-19 incidence changed regionally throughout the course of the U.S. pandemic, the potential COVID-19 impact on each racial and ethnic minority group also changed. During August 5–18, high COVID-19 incidence was reported by 2,034 (64.7%) counties, most of which were in the South (Figure 1). High COVID-19 incidence was reported by 92.4%, 74.5%, 65.9%, 64.7%, and 51.7% of counties with large Black, Hispanic, NH/PI, Asian, and AI/AN populations, respectively, during this period (Table) (Figure 2) (Supplementary Figure 2, https://stacks.cdc.gov/view/cdc/104229). During December 9–22, when 3,114 (99.1%) counties reported high COVID-19 incidence, >95% of counties with large populations of each racial and ethnic minority group reported high COVID-19 incidence (Table) (Figure 2) (Supplementary Figure 3, https://stacks.cdc.gov/view/cdc/104229).

## Discussion

Analysis of data from all 50 states and DC during April–December 2020 demonstrates how relative potential COVID-19 impact among racial and ethnic minority groups has changed during the U.S. COVID-19 epidemic. During the early weeks of April, a larger percentage of high-incidence counties was reported among those with large Asian populations and large Black populations, whereas during the early weeks of August, a larger percentage of high-incidence counties was reported among those with large Black populations and large Hispanic populations. By mid-December, high COVID-19 incidence was reported in nearly all counties.

As SARS-CoV-2 has spread throughout the United States, racial and ethnic minority populations have been profoundly affected. Previous CDC reports found that racial and ethnic minority groups were disproportionately represented among COVID-19 cases in counties with high or rapidly increasing incidence, and that these groups experienced higher COVID-19–associated hospitalization and death rates (*4*,*6*–*8*). Inequities in social, economic, and environmental conditions among racial and ethnic minority groups lead to disparities in health risks and outcomes (*9*), including those related to COVID-19.

This analysis and its population-based approach complements case-based analyses of racial and ethnic disparities. CDC continues to work with local and state health departments to improve reporting of race and ethnicity data for individual cases. Although case-based analyses might more directly assess these disparities, population-based approaches can illustrate the potential impact of COVID-19 across all racial and ethnic minority groups and across regions. This approach can also be used to examine potential disparities in other COVID-19–associated outcomes and behaviors, including vaccination administration.

State health departments can apply the approach taken in this study to analyze data for smaller geographic areas and identify when and where racial and ethnic minority groups might be experiencing high potential COVID-19 impact within their jurisdictions. Findings can be supplemented with analyses of indicators of social determinants of health, including occupation, health care access and utilization, income and wealth gaps, and housing stability and quality (*10*), to inform development of community engagement strategies that increase COVID-19 knowledge, testing, contact tracing, preventive care, vaccination administration, and disease management in populations at increased risk for COVID-19. In addition to examining counties with large racial and ethnic minority populations and high COVID-19 incidence, examining counties with large populations but low COVID-19 incidence might reveal lessons for effectively preventing the spread of COVID-19.

The findings in this report are subject to at least three limitations. First, counties might have large populations of more than one racial and ethnic minority group; therefore, comparisons across groups should be interpreted with caution. Second, a large racial and ethnic minority population within a county with high COVID-19 incidence does not necessarily mean that a disproportionate number of cases occurred among persons in that group. Analyses using case-level race and ethnicity data may be better suited to directly assess the disproportionate impact of COVID-19 on persons of color. Finally, the category indicating high 2-week COVID-19 incidence (>100 per 100,000) is conservative. The range for county-level incidence might be greater during periods of peak incidence (i.e., December) than during other periods.

CDC continues to collect data from local and state health departments to assess and monitor COVID-19 disparities and develop new ways to communicate data to the public and other partners.** These COVID-19 data^††^ can be examined by race and ethnicity and by the social determinants of health associated with these disparities to inform cross-sector programs and practices based on the CDC COVID-19 Response Health Equity Strategy.^§§^ Examining COVID-19 incidence in conjunction with race and ethnicity at the county level can identify areas where racial and ethnic minority groups might be experiencing high potential COVID-19 impact. CDC, as well as federal, state, and local partners, can use this approach throughout the COVID-19 response to direct public health activities intended to reach these areas. Current prevention measures, including correct and consistent use of masks, frequent handwashing, physical distancing,^¶¶^ avoiding crowds, limiting nonessential travel, and efforts to expand programs for vaccination, testing, screening, case investigation, case isolation, contact tracing and treatment can be integrated with longer-term community plans to alleviate long-standing health and social inequalities*** and improve health equity.

**Summary**

**What is already known about this topic?**

Long-standing systemic health and social inequities have placed many racial and ethnic minority groups at increased risk for COVID-19.

**What is added by this report?**

During April 1–14, 11.4% of counties reported high COVID-19 incidence, including 28.7% and 27.9% of counties with large Asian and Black populations, respectively. During August 5–18, this percentage was 64.7%, including 92.4% and 74.5% of counties with large Black and Hispanic populations, respectively. By December 9–22, 99.1% of counties reported high incidence.

**What are the implications for public health practice?**

As the COVID-19 pandemic evolves, public health efforts can be tailored to the needs of communities of color that may be experiencing high COVID-19 impact and integrated with longer-term plans to improve health equity.

## Footnotes

* Aggregate county-level case counts were downloaded through HHS Protect (accessed January 26, 2021).

†
https://www.census.gov/programs-surveys/popest.html

§
https://pro.arcgis.com/en/pro-app/help/mapping/layer-properties/data-classification-methods.htm

¶
https://www2.census.gov/geo/pdfs/maps-data/maps/reference/us_regdiv.pdf

**
https://www.cdc.gov/coronavirus/2019-ncov/community/health-equity/what-we-can-do.html

††
https://www.cdc.gov/coronavirus/2019-ncov/community/health-equity/racial-ethnic-disparities/index.html

§§
https://www.cdc.gov/coronavirus/2019-ncov/downloads/community/CDC-Strategy.pdf

¶¶
https://www.cdc.gov/coronavirus/2019-ncov/prevent-getting-sick/prevention.html

***
https://www.cdc.gov/minorityhealth/chdir/index.html
